# The morphology, morphometry and functionality of fresh and cryopreserved wisent (*Bison bonasus*) epididymal spermatozoa

**DOI:** 10.1038/s41598-023-40798-y

**Published:** 2023-08-24

**Authors:** Maria Eberhardt, Sylwia Prochowska, Agnieszka Partyka, Wiesław Bielas, Ann Van Soom, Wanda Olech, Wojciech Niżański

**Affiliations:** 1 Department of Reproduction and Clinic of Farm Animals, Faculty of Veterinary Medicine, Wroclaw University of Environmental and Life Sciences, Plac Grunwaldzki 49, 50-366 Wrocław, Poland; 2 Department of Internal Medicine, Reproduction and Population Medicine, Faculty of Veterinary Medicine, University of Ghent, Salisburylaan 133, 9820 Merelbeke, Belgium; 3 Department of Animal Genetics and Conservation, Institute of Animal Sciences, Warsaw University of Life Sciences, Ciszewskiego 8 St., 02-786 Warsaw, Poland

**Keywords:** Biodiversity, Animal physiology, Conservation biology

## Abstract

Epididymal spermatozoa obtained *post mortem* are considered a valuable source of genetic material which is often irrevocably lost. This makes these gametes constitute a key element in protection and restitution programs. The wisent (*Bison bonasus*, Linnaeus 1758) is a species that survived in zoos after extinction from its natural habitat. This resulted in a narrowing of the genetic pool of the whole population, which is at present derived from only 12 ancestors. Currently, wisent protection programs are aimed at preserving the genetic diversity by establishing a germplasm bank. The objective of this study was to comprehensively characterize the morphology, morphometry and functionality of wisent epididymal spermatozoa and evaluate the effectiveness of their cryopreservation in extender based on Tris buffer and chicken egg yolk. The median total number of spermatozoa obtained from one individual was 1985.0 × 10^6^ (62.5 × 10^6^–7452.0 × 10^6^). These gametes were characterized by median: 40.0% (0.5–70.0%) subjective motility, 69.8% (32.5–90.0%) viability and 54.3% (10.5–83.3%) normal morphology. The sperm head had a median size of 5.0 μm (3.5–6.7 μm) width, 8.5 μm (6.4–11.3 μm) length and 36.9 μm^2^ (23.7–48.6 μm^2^) surface area. The viable population of the obtained gametes was characterized by median values 53.2% (4.5–80.3%) of intact sperm membrane, 50.8 (26.0–76.6%) of intact acrosome, 0.4% (0–98.7%) of fragmented chromatin, 5.9% (0.0–88.8%) of cells with high mitochondrial potential and 42.1% (8.3–63.7%) without lipid peroxidation. The viable population of the frozen/thawed gametes was characterized by median values: 18.4% (2.4–57.9%) of intact sperm membrane, 35.1 (11.9–56.7%) of intact acrosome, 0.07% (0–89.2%) of fragmented chromatin, 12.8% (0.0–49.7%) of cells with high mitochondrial potential and 16.3% (2.2–53.6%) without lipid peroxidation. Due to the material originating from a relatively large number of wild individuals, the research presented here contributed to the description of certain species standards for the assessment of wisent epididymal spermatozoa. The presented effect of cryopreservation on these gametes justifies the use of an extender based on Tris buffer with the addition of chicken egg yolk. The obtained effects are satisfactory from the point of view of preserving valuable genetic material and their use in ART.

## Introduction

Gametes that have reached their final place of deposition—cauda epididymis, are equipped in a properly shaped acrosome and motility potential^1,2^. Retrieval and preservation of those spermatozoa gives opportunities to obtain offspring when ejaculation is no longer possible—in cases when orchiectomy is essential for an individual's health or sudden death^2,3^. The possibility of using epididymal spermatozoa has been proven in many domestic and wild species, resulting in these gametes being increasingly considered as a valuable source of genes which, in many cases, would otherwise be lost irrevocably^2,3^. In the case of endangered species, epididymal spermatozoa are often the only legal and ethical means of preserving genetic material from an individual. Therefore, the gametes obtained in this way constitute an invaluable treasure for protection and restitution programs^2,4^.

The wisent (*Bison bonasus*, Linnaeus 1758), otherwise known as the European bison, is a species that survived after extinction from the natural habitat^4^. Thanks to extensive international efforts, the wild population has been reestablished and the number of individuals has been growing constantly. In 2020 its status in the IUCN Red List was changed to “Near Threatened”^5^. However, this species has passed through the bottleneck. The entire population has only 12 ancestors, which resulted in a narrow genetic pool. Moreover, it is still a species with multiple small, fragmented populations^6^. This makes the wisent subject to inbreeding depression which can negatively affect birth weight and survival, reproduction, immunity to diseases, environmental stress and predation^7^. All of these factors carry risks not only for individuals, but for the entire herds. Currently, European bison protection programs are aimed at preserving valuable genes and slowing down the loss of biodiversity by establishing a wisent germplasm bank and working on implementation of Assisted Reproductive Techniques (ART) to avoid possible future inbreeding^4,5^.

Assisted reproductive techniques are a great achievement not only in the reproduction of livestock or companion animals, but also in the dynamically developing methods of wild species protection programmes^4^. However, to be successful in their implementation, it is necessary to thoroughly understand the reproductive physiology of a given species and the characteristics of its gametes. The possibility of using wisent epididymal spermatozoa collected post mortem and stored in liquid nitrogen for ART was published in several papers^8–11^. However, the comprehensive characterisation of the spermatozoa obtained from the wisent's epididymal tails with the basic methods, morphometry and flow cytometry has not been described thus far.

Fluorescence staining and flow cytometry assessment allow one to look at the structure of the sperm and thus make a functional assessment^12^. So far, the use of flow cytometry to characterise wisent spermatozoa has been described in one paper and involved only four individuals^10^.

The morphometric measurements of sperm heads have been demonstrated in males of many animal species not only mammals^13–16^ but also insects, fish^17^ and birds^18^. In stallions, boars and humans, the correlation between morphometric measurements and fertility has been described^17^. However, so far, the morphometry of sperm heads has been presented in only four wisent individuals^11^.

The objective of this study was to provide a comprehensive description of the morphology, morphometry and functionality of wisent epididymal spermatozoa. The material came from a relatively large group of wild individuals, which makes the obtained results a starting point for further research in the field of wisent reproduction. The second aim was to evaluate the effectiveness of cryopreservation of these gametes in the extender based on Tris buffer and egg yolk.

## Results

### Characteristics of wisent epididymal spermatozoa obtained *post mortem*

**Figure 1:**
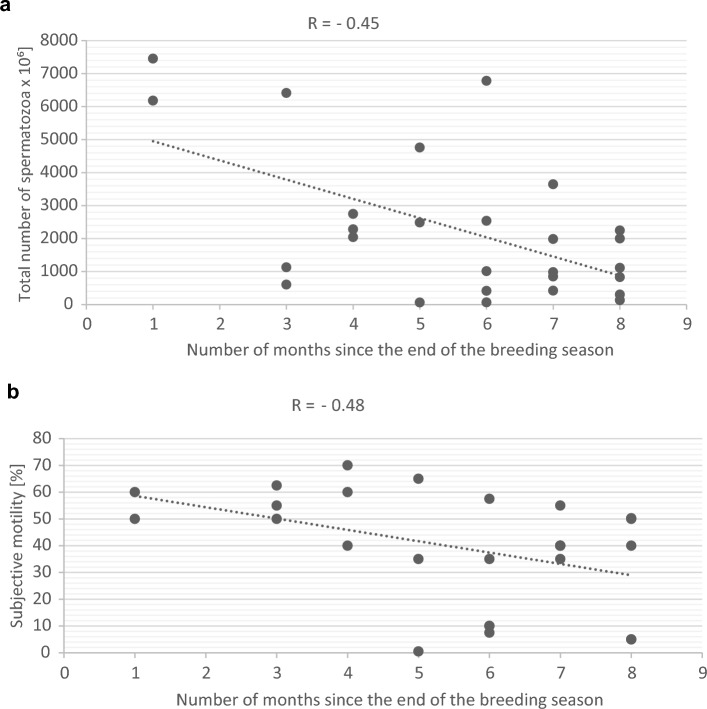
Results of Spearman's rank correlation coefficient between breeding season and sperm parameters: (**a**) total number of spermatozoa; (**b**) subjective motility, n = 27, *p* < 0.05.

The total sperm count, subjective motility, percentage of live and morphologically normal wisent spermatozoa obtained *post mortem* are presented in Table 1. There was huge inter-individual variability, which was related to the month of collection—significant moderate negative correlation was found for total sperm count and subjective motility, but not for the rest of assessed semen parameters. Similar correlations were not observed between semen parameters and animal age. Significant correlations are shown in Fig. 1. The total number of isolated spermatozoa and their subjective motility decreased with distance from the breeding season. Other Spearman correlation results can be found in the supplementary file.

**Table 1:** Characteristics of wisent epididymal spermatozoa collected post mortem, n = 27.

	The total number of spermatozoa [× 10^6^]	Subjective motility [%]	Live [%]	Normal morphology [%]
N	27	27	27	27
Min	62.5	0.5	32.5	10.5
Max	7452	70.0	90.0	83.3
Median	1985	40.0	69.8	54.3

Morphometric measurements of wisent epididymal spermatozoa heads are presented in Table 2.

**Table 2:** Morphometric measurements of wisent epididymal sperm heads—length, width, surface area. n = 1200 spermatozoa.

	Length [µm]	Width [µm]	Surface area [µm^2^]
N	1200	1200	1200
Min	6.4	3.5	23.7
Max	11.3	6.7	48.6
Median	8.5	5.0	36.9

The results of functional characteristics of wisent epididymal spermatozoa assessment by flow cytometry are presented in Table 3.

**Table 3:** The functional characteristics of wisent epididymal spermatozoa assessed by flow cytometry (n = 27).

	Live cells with intact sperm membrane [%]	Live cells with intact acrosome [%]	Cells with fragmented chromatin [%]	Live cells without lipid peroxidation [%]	Life cells with high mitochondrial activity [%]
N	27	27	27	27	27
Min	4.5	26.0	0	8.3	0
Max	80.3	76.6	98.7	63.7	88.8
Median	53.2	50.8	0.4	42.1	5.9

### Evaluation of the effectiveness of cryopreservation of wisent epididymal spermatozoa in the extender based on Tris buffer and egg yolk

**Figure 2:**
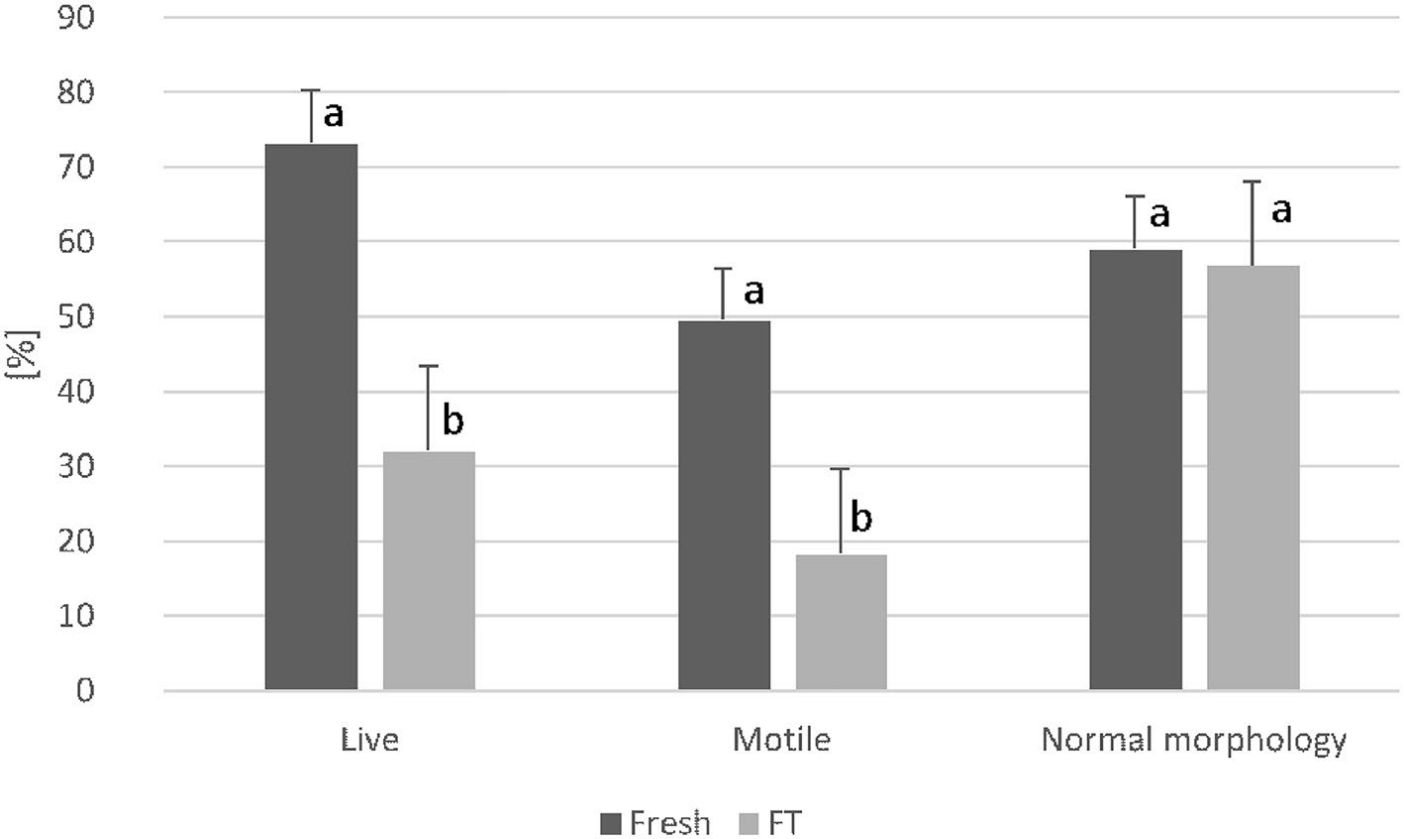
The comparison of viability, motility and morphology of spermatozoa before (fresh) and after cryopreservation (FT), data presented as mean mean ± SE; (**a**, **b**) within each parameter are significantly different (*p* < 0.05) (n = 20).

Cryopreservation significantly lowered the percentage of live and motile spermatozoa. However, this process had no significant effect on the morphology of the gametes. The comparison of results of the basic evaluation of wisent epididymal spermatozoa before and after cryopreservation are presented in Fig. 2.

**Figure 3:**
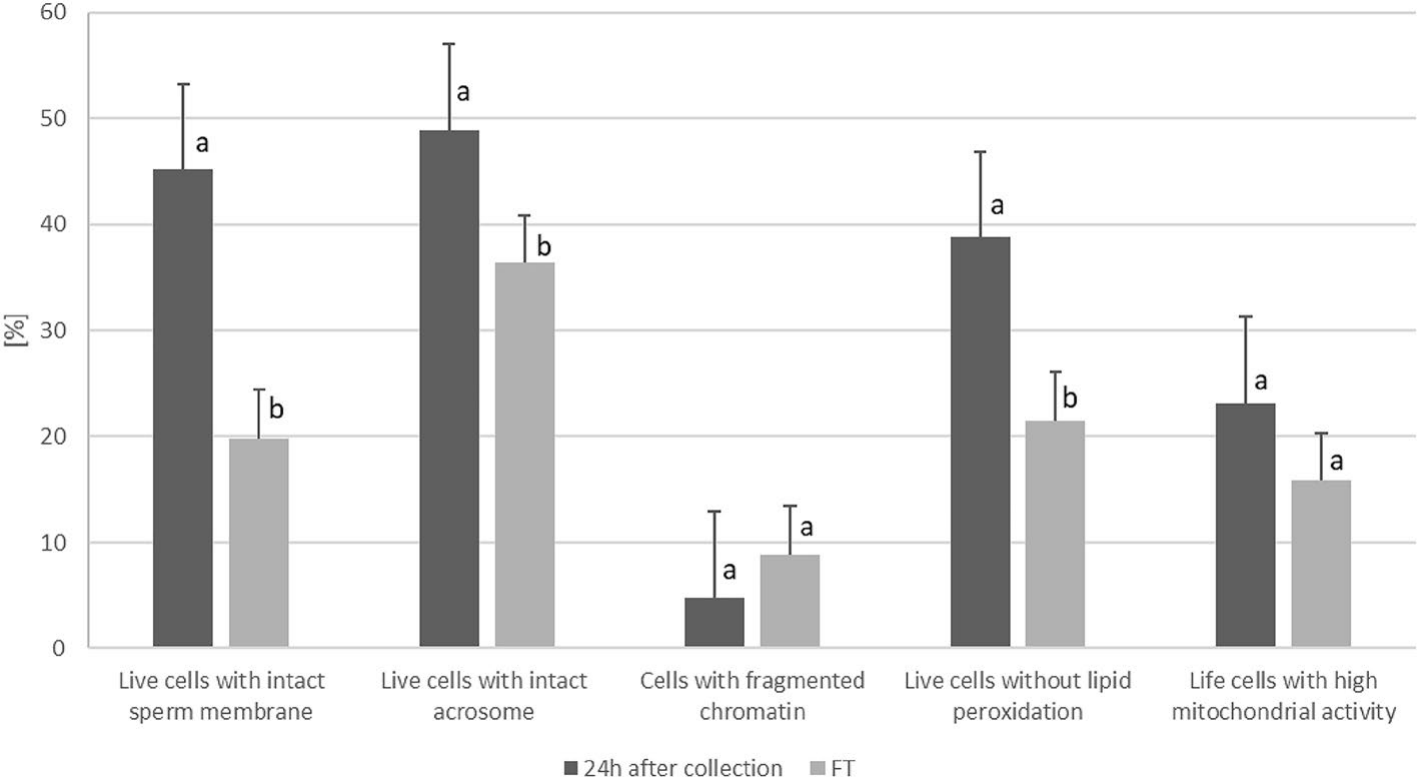
The comparison of functional characteristics of wisent spermatozoa 24 h from collection and after cryopreservation (FT) assessed by flow cytometry. Data presented as mean ± SE; (**a**, **b**) within each parameter are significantly different (*p* < 0.05) (n = 20).

The comparison of obtained results of functional spermatozoa assessment after freezing and thawing process by flow cytometry with initial values (24 h after collection) are presented in Fig. 3. Among the parameters assessed by flow cytometry, significant differences before and after thawing were found for the percentage of live spermatozoa characterised by: intact sperm membrane, intact acrosome, and presence of lipid peroxidation (*p* < 0.05). There were no significant differences in the percentage of spermatozoa characterised with high mitochondrial potential and fragmented chromatin before and after the cryopreservation process.

## Discussion

Epididymal sperm is a treasure for the protection of biodiversity in the wildlife world. In the case of wisent, it is the only way to preserve genes that would be otherwise lost with the death of an individual. However, in order to fully use this hidden potential, it is necessary to expand knowledge in this area. Although the European bison is one of the well-known protected animals, many aspects of its reproduction have still not been described. In the available literature, the process of spermatogenesis in this species is described in detail, however the male gametes of wisent have still not been fully characterised. Therefore, this article presents the properties of wisent epididymal spermatozoa such as morphology, morphometry and functionality—for the first time characterised on the basis of material obtained from such a vast number of wild individuals. Due to the mentioned lack of information regarding European bison, the authors of this article compared obtained results with other representatives of the bovidae family: Cattle (*Bos taurus*) and two subspecies of the American bison—Plains bison *(Bison bison bison)* and Wood bison *(Bison bison athabascae).*

The median amount of gametes obtained from the wisent individual was 1985 × 10^6^ ranged from 62.5 × 10^6^ to 7452 × 10^6^ which is higher than results observed in the Plains bison which range from 146 to 830 × 10^6^^19^ and cattle bulls results, which ranged from 440 × 10^6^ to 1100 × 10^6^^20,21^ The median percentage of live spermatozoa assessed by the eosin dye was on 69.8%, which is lower than results observed in domestic cattle bull epididymal spermatozoa—86.2%^22^. The obtained wisent spermatozoa were characterised by 40% (0.5–70.0%) median motility which is lower in comparison to the motility of Plains bison which was evaluated as 71.7%^23^–78.0%^24^ and Wood bison—62.0%^24^. The subjective motility was also lower than this evaluated for cattle epididymal sperm which ranges from 64.4^20^ to 80.0%^21^.

The median percentage of morphologically normal spermatozoa obtained from wisents epididymides was 54.3% ranging from 10.5 to 83.3%. Similar results were obtained in American bison when the average percentage of normal sperm was ranging from 56.8 ± 16.0 to 70.7 ± 19.4%^25^.

Morphometric measurements showed that the head of wisent spermatozoa is characterised by median: width 5.0 μm (3.5–6.7 μm), length 8.5 μm (6.4–11.3 μm) and an area 36.9 μm^2^ (23.7–48.6 μm^2^). The obtained results are adequate to the previously described measurements on a smaller number of wisent specimens (n = 4)^11^. Measured dimensions are similar in both subspecies of the American bison^26^. Spermatozoa head parameters measured in wisents like: width and area are greater than in cattle bull gametes^27^ and smaller in case of head length. Morphometric measurements using the computer-assisted sperm morphometry analysis (CASMA) system have been performed in many animal species^11,13,14,17–19,26,28^. The main goal of the cited research is to find a correlation between the sperm heads morphometry and male fertility. The relationship between fertility and morphometry has been proven in red deer^29^ and ram^30,31^. Similar relationships could not be proven in studies on tomcat urethral semen^14^.

The obtained results of manual measurements may be the starting point for similar research on European bison. However, in order to fully use the potential of morphometric measurements and relate them to other sperm assessment parameters, it is necessary to continue research using CASMA systems and to separate sperm subpopulations using it.

Flow cytometry assessment revealed that samples obtained from wisent epididymides are characterised by median 53.2% of spermatozoa with intact sperm membrane which is lower than in bull epididymal sperm^32^.

The percentage of spermatozoa with an intact acrosome was also lower than that of domestic cattle bull^32^. However, in the cited article, the authors describe that the material was being prepared for analysis within 2 h after collection^32^, the longer period between collection and evaluation may be one of the reasons for the described differences in spermatozoa parameters.

The results of the assessment by flow cytometry were worse compared to those previously described by the authors^10^. However, in the cited study, the spermatozoa were qualified for cryopreservation on the basis of subjective mobility, eliminating the samples with inferior quality, which possibly explains the differences in the mean percentages of some parameters.

When considering the presented results, it should be taken into account that all gametes used in this study were collected immediately after the death of the individuals, but each eliminated animal was qualified for it due to serious injury, illness or great age. This may be the reason for differences in the quality between the epididymal spermatozoa of the wisent and other species, the epididymides of which were obtained in abattoirs during technological slaughter. What is more, the material used in the present study was obtained outside the breeding season, which may also affect the parameters of the isolated gametes.

The second aim of the presented research was to evaluate the results and experience gained during freezing wisent spermatozoa from 20 living in the wild individuals.

The greatest drawback in research on the wild animals reproductive physiology is a low availability of biological material. This creates limitations in adaption ART in those species. Hence appears the need to use material coming from closely related non endangered species as a model for selecting the best protocol for evaluation and dealing with gametes collected from wild individuals^33^. The limited number and great value of the collected material did not allow for studying different extenders. For this reason, it was decided to use an extender with proven usefulness described in publications on the freezing of semen of other members of the Bovidae family^28,32–34^ and had been previously checked in a smaller number of wisent individuals^9^. According to the authors' knowledge, the results presented in this study are the first such comprehensively describing the effects of cryopreservation of wisent epididymal spermatozoa. The authors made an attempt to adapt well-known methods that are part of the gold standard in animal semen evaluation to this wisent population. This makes the presented research somewhat pioneering, but also poses many difficulties in assessing these gametes due to the small amount of information described so far in this species.

Particularly noteworthy are the significant differences between fresh and thawed samples in the percentage of spermatozoa with an intact sperm membrane, due to its importance for the proper functioning of the entire cell related to the viability, motility and capacity to interact with the extracellular environment^12^. These differences ranged from 4.50 to 63.76%. That could have also influenced the loss of the percentage of motile spermatozoa observed in examined samples which ranges from 0.00 to 59.00%. However, the average total motility after thawing was similar to the result from the pilot study on the use of wisent epididymal sperm to obtain hybrids with domestic cattle by artificial insemination^9^. The obtained subjective motility was lower than the percentage observed in Plains bison and Wood bison epididymal spermatozoa frozen in Tris and egg yolk containing extender^18^.

To fulfill its role, spermatozoa have to be equipped with a properly built acrosome allowing its cargo to penetrate the zona pellucida^12^. For this reason, acrosome integrity is the important feature assessed during spermatozoa evaluation^12^. In the present study, the deterioration of the percentage of sperm with an intact acrosome was observed. Losses during the freezing/thawing process ranged from 0.36 to 40.34% and were lower in comparison to the level of those related to sperm membrane integrity. Nevertheless, those changes were not surprising and were also observed in thawed epididymal sperm of domestic cattle^32^.

The cryopreservation process had no significant impact on the chromatin status. What’s worth noticing, even after freezing–thawing DFI % remained on relatively low level, which is promising in the context of the use of these spermatozoa for ART.

When assessing the effectiveness of the cryopreservation of wisent epididymal spermatozoa, certain limitations should be taken into account, which are related both to the origin of the sperm, the method of its collection and the process of freezing / thawing itself. Significant limitation to the final effects of cryopreservation of the described sperm is their origin. Although the possibility of the effective use in ART of epididymal sperm has been described in many species of farmed and wild animals, it has also been shown that in comparison with ejaculated semen there seems to be a difference in sperm movement characteristics lower velocity, less straightness and linearity^3,32^. In addition, the incision method used to collect epididymal sperm favours the contamination of the samples with tissue detritus, which may additionally reduce the post-freezing quality of the spermatozoa^10^.

All these factors can exacerbate the damage that results from the stress generated during the freezing process itself, i.e. cold shock, osmotic and toxic stresses connected to the exposure to cryoprotectants and ice formation^12^ and results in impaired fertility by comparison with fresh semen. The reduction arises from both a lower viability post-thaw and sublethal dysfunction in a proportion of the surviving subpopulation^35^. This was clearly visible in obtained results where in all evaluated parameters similar deterioration was observed except percentage of: morphologically normal, damaged chromatin and with high mitochondrial potential spermatozoa.

Another adversity faced by male gametes and basically every living cell is oxidative stress, which can be described as an imbalance between the production of reactive oxygen species (ROS) and the possibility of their neutralisation by antioxidants. However, it is noteworthy that the right amount of them is crucial in the process of capacitation, acrosomal reaction and fusion with the oocyte^12^. Nevertheless, the disturbance of the aforementioned balance and thus the excess of ROS leads to the destruction of nucleic acids, proteins, lipids and carbohydrates, ultimately leading to the cell’s death^36^ and might lead to lowered male fertility^12^. Spermatozoa are particularly exposed to oxidative stress during the cryopreservation process which certainly contributed to the worse results of the parameters tested compared to the initial values^36–38^. Immature and morphologically abnormal spermatozoa and seminal leukocytes are the main sources of ROS in ejaculates^12^. For this reason, even if it was not evaluated in this study, it should be taken into consideration that cryopreserved wisent spermatozoa were already initially characterised by an increased percentage of immature and morphologically abnormal spermatozoa and the presence of leukocytes due to the above-mentioned origin of gametes. All this together could be the reason for the increased percentage of lipid peroxidation in the assessed spermatozoa.

Cryopreservation, despite its many undeniable advantages, is an invasive process for gametes^35^. The described changes in European bison spermatozoa were not surprising because they result from the specificity of the process itself and have been extensively described in many species of animals, including other representatives of the Bovidae family^28,32–35^. In addition, obtaining sperm from the epididymis of sick and eliminated animals could have further limited its quality. To the knowledge of the authors, there are no minimum requirements for epididymal sperm as there are for ejaculated semen. However, when assessing frozen spermatozoa as a source of genes that would be irretrievably lost, the number of gametes obtained from each of the preserved epididymides is sufficient to be used in ART.

## Conclusions

The research carried out, due to the material from a relatively large number of individuals of protected species, contributed to the description of certain set points for the assessment of the quality of samples available for banking. To our knowledge, this is the first study that characterises wisent epididymal spermatozoa in such a broad way, consequently increasing the general knowledge about this protected species.

In research on maintenance of biodiversity, all preserved genetic material is invaluable. The obtained results of cryopreservation of wisent epididymal spermatozoa with the use of an extender based on Tris buffer with the addition of chicken egg yolk allowed to preserve the genetic material of all individuals in a quality sufficient for use in the Assisted Reproductive Technology. Nevertheless, research on their preservation should be continued to fully exploit their great, yet still hidden potential.

## Materials and methods

### Chemicals and media

All reagents and extender components were purchased from Sigma- Aldrich (St. Louis, MO, USA).

The fluorescence probes were purchased from Life Technologies Ltd., Grand Island, NY, USA.

Animals.

The material for the research was collected post-mortem from 27 individuals at age from 3 to 20 years. The wisents came from captive breeding herds or show enclosures from 13 locations in Poland. Collections were performed outside of the breeding season (September–April) between the years 2015–2023.

No animal was killed for the purposes of this study—all animals were eliminated due to poor health status, diagnosed infectious disease or aggressive behaviour under the permits issued by the General Director for Environmental Protection. Local Ethics Committee approval was not required.

### Spermatozoa collection

Epididymides were collected immediately after the animal's death. Epididymal tails were dissected, cleaned from connective tissues and blood vessels, then placed on glass Petri dishes containing 4 ml of a Tris-based extender (Tris (hydroxymethyl)- aminomethane (0.2 M), citric acid monohydrate (0.06 M), glucose (0.05 M), distilled water up to 100 ml) (30 °C) and sliced with scalpel blade. Subsequently, dishes were left on the warming platform for 10 min of incubation before initial analysis.

### Sperm analysis

#### Basic assessment

Concentration of spermatozoa was assessed in Thoma chamber (× 400) using the phase contrast microscope (Nicon Eclipse E200). Volume of sperm suspension was measured by an automatic pipette. Total sperm count was calculated by multiplying sperm volume and concentration.

Motility of spermatozoa was assessed subjectively using the phase contrast microscope (Nicon Eclipse E200) with a warming stage by placing 10 μL of sample on the glass slide and covered with a cover slip (×400).

**Figure 4:**
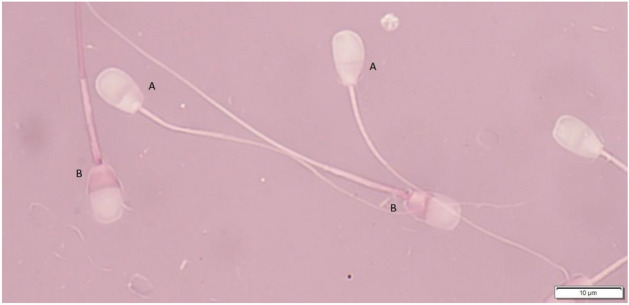
Evaluation of the percentage of live and dead spermatozoa using the eosin-nigrosin dye. (**A**) unstained viable spermatozoa; (**B**) stained dead spermatozoa.

The percentage of live and dead spermatozoa was evaluated on eosin-nigrosine stained smears. Ten microliters of sperm sample were mixed with 10 mL of eosin–nigrosin solution, spread onto a microscope slide and dried on air. The slides were examined under the light microscope (magnification ×1000). Two hundred spermatozoa were evaluated per each slide. Pink-stained spermatozoa were considered dead. Gametes that remained unstained were classified as live (Fig. 4).

Morphology of spermatozoa was evaluated on smears stained with Bydgoska method^32^. The percentage of presence of proximal droplet, distal droplet, head shape abnormalities, damaged acrosome, midpiece defects, dag- like defect, bent tail, detached head and coiled tail was evaluated in 200 of spermatozoa per each slide. Spermatozoa which did not show these defects were described as morphologically normal.

#### Sperm head morphometry

**Figure 5:**
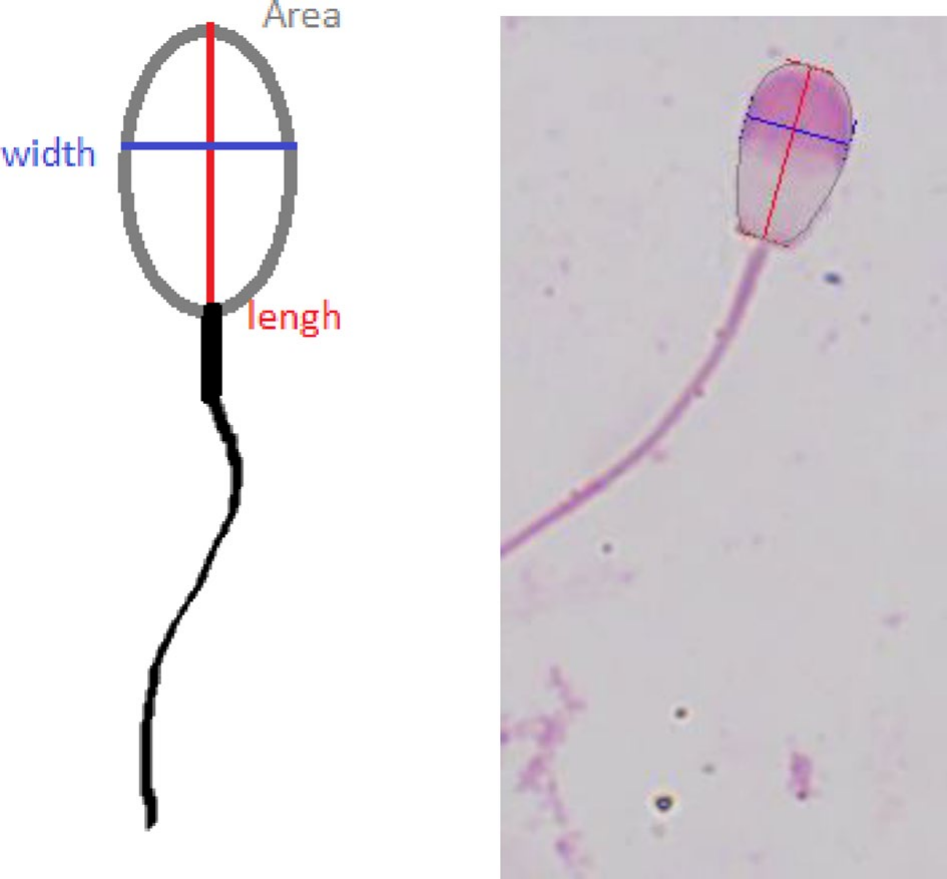
Sperm head morphometry measurements.

Sperm head morphometry was evaluated with the use of cellSens Dimension Software (Olympus Europa SE & Co. KG). The length, width and area of head were measured manually on smears stained with Bydgoska method^39^. For morphometric measurements, slides from best quality samples were selected. In total, 1200 spermatozoa were measured. The method of making measurements was presented in Fig. 5.

### Flow cytometry assessment

All tests were performed using Guava EasyCyte 5 (Merck KGaA. Darmstadt. Germany) cytometer. Gametes acquisitions were analysed with the GuavaSoft™ 3.1.1 software (Merck KGaA. Darmstadt. Germany). The fluorescent probes were excited by an argon ion 488 nm laser. The non-sperm events were gated out based on scatter properties and not analysed. A total of 10.000 events were analysed for each sample. All stainings and analysis were performed following the protocol used in our laboratory, which was described before for other animal species^36,40,41^  and which was proven suitable for wisent^10^.

The spermatozoa membrane integrity was assessed by using SYBR-14 stain combined with propidium iodide (PI). 300 μL of sample was incubated in the dark for 10 min with 5 μL of SYBR-14 working solution (0.1 μL SYBR14 + 4.9 μL TRIS based extender). The analysis was performed after 3 min of incubation with 3 μL of PI. Spermatozoa showing green fluorescence were classified as those with intact membranes and those with red fluorescence were classified as dead^10,40,41^.

Acrosome integrity was assessed by lectin PNA stain from Arachis hypogaea Alexa Fluor® 488 conjugate combined with propidium iodide (PI). Diluted samples were mixed with 10 μL of PNA working solution (1 μg/mL) and incubated for 5 min at room temperature in the dark. Subsequently, the samples were washed and 3 μL of PI was added. Spermatozoa showing green fluorescence were classified as those with damaged acrosome^10,40,41^.

Mitochondrial activity was assessed using the JC-1 dye combined with propidium iodide (PI). 500 μL of samples were stained with 0.67 μL JC-1 stock solution (3 mM stock solution of JC-1 in DMSO). Subsequently, the samples were incubated for 20 min at 37 °C in the dark. After incubation 3 μL of PI was added. Sperm emitting orange fluorescence were classified as high mitochondrial membrane potential. Spermatozoa emitting green fluorescence were classified as having low mitochondrial potential^10,40,41^.

Lipid peroxidation was assessed with C11-BODIPY581/591 probe combined with propidium iodide (PI). 1 μL of 2 mM C11-BODIPY581/591 in ethanol was added to the diluted samples and incubated for 30 min at 37 °C in the dark. Subsequently, samples were centrifuged at 500×*g* for 3 min. The sperm pellets were resuspended in 500 μL of TRIS based extender. To determine viability, the spermatozoa were stained with PI and incubated for 3 min at room temperature. Sperm emitting only orange fluorescence were considered live without lipid peroxidation-LPO (L/LPO-)^10,40,41^.

To determine chromatin status the acridine orange (AO) dye was used. 100 μL samples were subjected to brief acid denaturation by adding 200 μL of the lysis solution (Triton X-100 0.1% (v/v), NaCl 0.15 M, HCl 0.08 M, pH 1.4). After 30 s, 600 μL of AO solution (6 μg AO/mL in a buffer: citric acid 0.1 M, Na2HPO4 0.2 M, EDTA 1 mM, NaCl 0.15 M, pH 6) was added. The analysis was performed after 3 min of incubation. Spermatozoa with normal DNA configuration were emitting green fluorescence. Cells showing red fluorescence were considered as those with denatured DNA^10,40,41^. Spermatozoa cryopreservation.

After basic quality assessment, samples from 20 individuals, characterised by a minimum of 35% percent subjective motility and a minimum of 120 × 10^6^ total sperm count, were qualified for freezing.

Subsequently, concentration of spermatozoa and subjective motility were evaluated as it was described above.

Suspension of spermatozoa was diluted with the extender based on Tris buffer and egg yolk (Tris (hydroxymethyl)- aminomethane (0.2 M), citric acid monohydrate (0.06 M), glucose (0.05 M), egg yolk (20% v/v), penicillin (5000 IU) streptomycin (100 mg) and distilled water up to 100 ml) at 22 °C to obtain the concentration 200 × 10^6^/ml.

After first dilution, samples were placed in a water bath and cooled down to 5 °C in the refrigerator. When the required temperature was reached, the second dilution was performed by adding extender as above with an addition of glycerol to obtain final sperm concentration 160 × 10^6^/ml and final glycerol concentration 6%.

Diluted samples were left for 90 min equilibration at 5 °C. Subsequently, the suspensions of spermatozoa were loaded into 0.25 ml straws (40 × 10^6^ spermatozoa per straw). The free end of the straw was closed with polyvinyl alcohol (PVA). Straws were placed in the liquid nitrogen vapours for 15 min and then immersed in the liquid nitrogen and stored^10^.

To assess the effectiveness of the cryopreservation process, one straw from each sample was thawed (water bath, 37 °C, 30 s) from 1 week to 1 month from the day of collection. Post-thaw quality was evaluated by the same methods as were described above.

### Statistical analysis

Shapiro–Wilk’s test was used to assess data normality and because majority of date were not normally distributed, the results of the assessment of sperm characteristics were presented in the form of mean, mean with standard error, median, as well as minimum and maximum values calculated by using PAST 4.03 program^42^.

Nonparametric test (Wilcoxon signed ranks test) were used to evaluate differences between fresh and frozen thawed samples. Differences were considered significant at *p* ≤ 0.05. Spearman correlation was calculated between the month of collection and semen parameters. September, considered the end of the breeding season in wisents, was adopted as month No. 1. Spearman correlation was also calculated between the animal age and semen parameters.

Statistical analyses were performed by using R Programming Language^43^.

### Ethical approval and consent to participate

The authors declare that during the work, no animal studies were conducted and no animals were shot on purpose of those studies. Samples were collected post-mortem from individuals shot under the permits issued by the General Director for Environmental Protection. Local Ethics Committee approval was not required.

A permit issued by the Regional Director for Environmental Protection in Wrocław (WPN.6401.170.2019.MH) for the possession and keeping of Bison bonasus cells, sperm, oocytes, and fibroblasts at the Bison Sperm Bank located in the building of the Department of Reproduction with the Clinic of Farm Animals at 49 Grunwaldzki Square, Wrocław, Poland.

## Data availability

The datasets used during the current study are available from the corresponding author on reasonable request.

### Supplementary Information

Supplementary Information.

## Abbreviations

AO: Acridine orange dye
ART: Assisted reproductive techniques
FT: Freezing/thawing; frozen/thawed
IUCN: The International Union for Conservation of Nature
PI: Propidium iodide
PVA: Polyvinylpyrrolidone
ROS: Reactive oxygen species
