# Supplementary material for: The morphology, morphometry and functionality of fresh and cryopreserved wisent (Bison bonasus) epididymal spermatozoa
Source: Sci Rep. 2023 Aug 24;13:13866. doi: 10.1038/s41598-023-40798-y (PMC10449768; doi:10.1038/s41598-023-40798-y)
Supplement: Supplementary file 1 — Supplementary Information. [file 41598_2023_40798_MOESM1_ESM.docx]

Supplementary material

Figure 1. Results Spearman's rank correlation coefficient between breeding season and sperm parameters: 1a- total number of spermatozoa; 1b- subjective motility; 1c- viability; 1d- normal morphology; 1e- live cells with intact sperm membrane; 1f- live cells with intact acrosome, 1g- cells with fragmented chromatin,1 h- live cells without lipid peroxidation; 1 i- live cells with high mitochondrial potential

1 a)

1 b)

1 c)

1 d)

1 e)

1 f)

1 g)

1 h)

1 i)

Table 1. p values of the Spearman rank correlation coefficient between breeding season and semen parameters. Significant correlations are marked with an asterisk (p<0.05).

|  | Total number of spermatozoa [x10⁶] | Subjective motility [%] | Viability [%] | Normal morphology [%] | Live cells with intact sperm membrane [%] | Live cells with intact acrosome [%] | Live cells with fragmented chromatine [%] | Live cells without lipid peroxidation [%] | Live cells with high mitochondrial potential |
| --- | --- | --- | --- | --- | --- | --- | --- | --- | --- |
| p value | 0.02 * | 0.01 * | 0.70 | 0.05 | 0.54 | 0.60 | 0.29 | 0.82 | 0.11 |

Table 2. r values ​​of the Spearman's rank correlation coefficient for age and individual sperm parameters. No significant correlation were found (p>0.05).

|  | Total number of spermatozoa [x10⁶] | Subjective motility [%] | Viability [%] | Normal morphology [%] | Live cells with intact sperm membrane [%] | Live cells with intact acrosome [%] | Live cells with fragmented chromatine [%] | Live cells without lipid peroxidation [%] | Live cells with high mitochondrial potential |
| --- | --- | --- | --- | --- | --- | --- | --- | --- | --- |
| R value | 0.181798 | - 0.106414 | - 0.067831 | - 0.021569 | 0.216309 | 0.015407 | - 0.110379 | 0.075392 | 0.159402 |
